# Gαq protein signaling in the bed nucleus of the stria terminalis regulate the lipopolysaccharide-induced despair-like behavior in mice

**DOI:** 10.3934/Neuroscience.2020027

**Published:** 2020-11-10

**Authors:** Nao Fukuwada, Miki Kanno, Satomi Yoshida, Kenjiro Seki

**Affiliations:** Department of Pharmacology, School of Pharmaceutical Science, Ohu University, 31–1 Misumido, Tomitamachi, Koriyama, Fukushima 963–8611, Japan

**Keywords:** α_1_-adrenergic receptor, Gαq protein signaling, lipopolysaccharide, despair-like behavior, the bed nucleus of the stria terminalis

## Abstract

Major depressive disorder (MDD) is highly comorbid with anxiety disorders. It has been reported that the bed nucleus of the stria terminalis (BNST) is important for the induction of anxiety and MDD. Recently, the Gαq protein signaling within the BNST is involved in the induction of anxiety through Gαq protein signaling-mediated RNA-editing of GluR2 subunit, which produces the calcium (Ca^2+^)-impermeable α-amino-3-hydroxy-5-methyl-4-isoxazolepropionic acid (AMPA) receptor. On the other hand, the role of Gαq protein signaling within the BNST on the induction of MDD has never been reported yet. Therefore, we investigated whether Gαq protein signaling-producing the Ca^2+^-impermeable AMPA receptors in the BNST is involved in the lipopolysaccharide (LPS)-induced depressive-like behavior, particularly, despair-like behavior. When mice were systemically challenged with a single dose of LPS (1.2 mg/kg, i.p.), the immobility time during tail suspension test (TST) was increased 24 h after LPS injection. However, pretreatment with bilateral intra-BNST injection of neomycin (6.5 mM, 0.125 µL/side), an inhibitor of phospholipase C that is activated by Gαq protein-coupled receptor stimulation, extended the LPS-induced increase in the immobility time of TST. Furthermore, the co-pretreatment with bilateral intra-BNST injection of neomycin with 1-naphthylacetyl spermine (3 mM, 0.125 µL/side), an antagonist of Ca^2+^-permeable AMPA receptor, to mimic one of the final forms of Gαq protein activation, abolished the aggravated effect of neomycin and significantly shortened the immobility time compared with the control mice with an intra-BNST injection of artificial cerebrospinal fluid before LPS injection. However, pretreatment with bilateral intra-BNST injection of MDL-12,330A (10 µM, 0.125 µL/side), an inhibitor of adenylyl cyclase that is activated by Gαs protein-coupled receptor stimulation, did not affect the LPS-induced increase in the immobility time of TST. These results indicate that the Gαq protein signaling-mediated RNA-editing of GluR2, which produces the Ca^2+^-impermeable AMPA receptors within the BNST, regulates the LPS-induced despair-like behavior.

## 1. Introduction

Approximately 85% of patients with major depressive disorder (MDD) also have comorbid anxiety disorder (AD) [1], although anxiety and depression have been considered as two distinct entities according to the diagnostic criteria [2]. Recently, it has been reviewed by Liu et al that the either MDD or ADs might increase the risk for the development of other disease [3]. The role of the bed nucleus of the stria terminalis (BNST) in anxiety disorders has been well investigated [4],[5],[6]. On the other hand, the role of BNST on the induction of MDD has not been well studied yet to date. Recent clinical studies demonstrated that the electrostimulation of the BNST ameliorates the MDD in human [7],[8] and rats [9]. The lesion of the BNST in rats has been shown to enhance the induction of learned despair [10],[11]. These results indicate that the inactivation of BNST might contribute to the induction of MDD. In contrast to these, some rodent studies suggest that the acute reversible inactivation of the BNST induces antidepressant-like effects [12],[13]. Furthermore, a high c-fos expression is observed in the BNST following systemic immune challenge with lipopolysaccharide (LPS), which has been used for inflammation-induced depression-like behavior in rodents [14],[15],[16]. These results suggest that the BNST activation contributes to the induction of despair-like behavior of MDD. These contradictions complicate our understanding the role of BNST on the induction of MDD. Therefore, it is important to investigate how BNST contribute to the induction of MDD.

Chronic treatment with selective serotonin reuptake inhibitors (SSRI), which are used for MDD, up-regulates the serotonin (5-hydroxytryptamine; 5-HT) 5-HT_2_ receptor subtypes and α_1_-adrenergic receptors in the cortical region [17],[18] but down-regulates the expression level of 5-HT_2_ receptors in the amygdala, when the anxiolytic effect of SSRI is available [19]. In agreement, ACH-000029, an antagonist of 5-HT_2A_ and α_1_-adrenergic receptors and partial agonist of 5-HT_1A/D_ receptors, reduced c-fos expression in the amygdala and BNST following single prolonged stress [20]. 5-HT_2_ serotonergic receptors and α_1_-adrenergic receptors are both coupled with Gαq protein. Activation of Gαq-mediated signaling in the BNST induces anxiety-like behavior [21], indicating that the inactivation of the Gαq protein in the BNST may contribute to the therapeutic effect for anxiety. However, the role of the Gαq protein in the BNST on the induction of MDD has not been reported. Therefore, it is important to investigate whether the activation of the Gαq protein in the BNST contribute to the induction of MDD.

With the consideration that the noradrenergic stimulation elicits stress responses [22], stress may be mediated, in part, by noradrenergic inputs to the paraventricular nucleus from the locus coeruleus and the medullary A1 and A2 nuclei [22],[23]. Noradrenergic receptors in the BNST are mainly innervated by the brainstem A1 and A2 noradrenergic neurons [24]. Interestingly, within the BNST, it has been demonstrated that the different forms of long-term synaptic depression (LTD) could be induced by Gαq protein activation. For example, restraint stress, which could induce the depressive-like behavior in both the mice and rat [25], disrupts the α_1_-adrenergic receptor-dependent LTD, which is induced by an increase in the calcium (Ca^2+^)-impermeable α-amino-3-hydroxy-5-methyl-4-isoxazolepropionic acid (AMPA) receptors through the catalysis of RNA editing by adenosine deaminases that act on RNA2 (ADAR2) [21],[26]. These results indicate that the α_1_-adrenergic receptor-dependent LTD is due to the induction of calcium (Ca^2+^)-impermeable AMPA receptor, may contribute to the induction of MDD in mice through the Gαq protein signaling-mediated RNA-editing of GluR2. On the contrary, Gαq protein-coupled group 1 metabotropic glutamate receptor (mGluR)-dependent LTD in the BNST required the endocytosis of AMPA receptors, indicating that some different forms of LTD could be induced by activation of the Gαq protein in the BNST.

The present study investigated the effect of Gαq protein-coupled receptor stimulation-mediated signaling in the BNST on the LPS-prolonged the immobility time during the tail suspension test (TST), a phenomenon that reflects the learned despair-like behavior, which is one of the depressive-like behaviors in rodents.

## 2. Methods and materials

### 2.1. Ethics statement, animal care, and LPS injection

As we have reported that the ethics and animal care in our University [27], this study was also approved by the Animal Care Committee of Ohu University (Nos. 2016–29, 2017–34, 2018–29, and 2019–39). We performed our animal experiments according to the guideline of Animal Care Committee of Ohu University, which complies with the criteria mandated by the Japanese Law for the Humane Treatment and Management of Animals. Principles of laboratory animal care were followed, with special care taken to minimize animal distress and utilize the minimum number of animals needed for all experiments, under the principle of the three Rs (Replacement, Reduction, and Refinement) [28]. Adult male ICR mice (7–12 weeks old) were supplied by Charles River Laboratories (Yokohama, Japan) and CLEA Japan, Inc. (Tokyo, Japan). All mice were housed at 25 ± 2 °C on a 12-h light (08:00 to 20:00 h)/12-h dark (20:00 to 08:00 h) cycle and were supplied *ad libitum* access to food and water. This study was approved by the mice intended for guide cannula implantation into the skull were handled individually once daily for the 4 days before surgery, and then twice daily during the 2 days after recovery from anesthesia until the behavioral tests were performed. LPS was dissolved in sterile endotoxin-free isotonic saline and administered intraperitoneally on the first day at a dose of 1.2 mg/kg which dose is an adequate range for inducing the LPS-induced depressive-like behaviors [16],[29],[30],[31].

### 2.2. Guide cannula implantation and intra-BNST drug injections in awake mice

**Figure 1.**
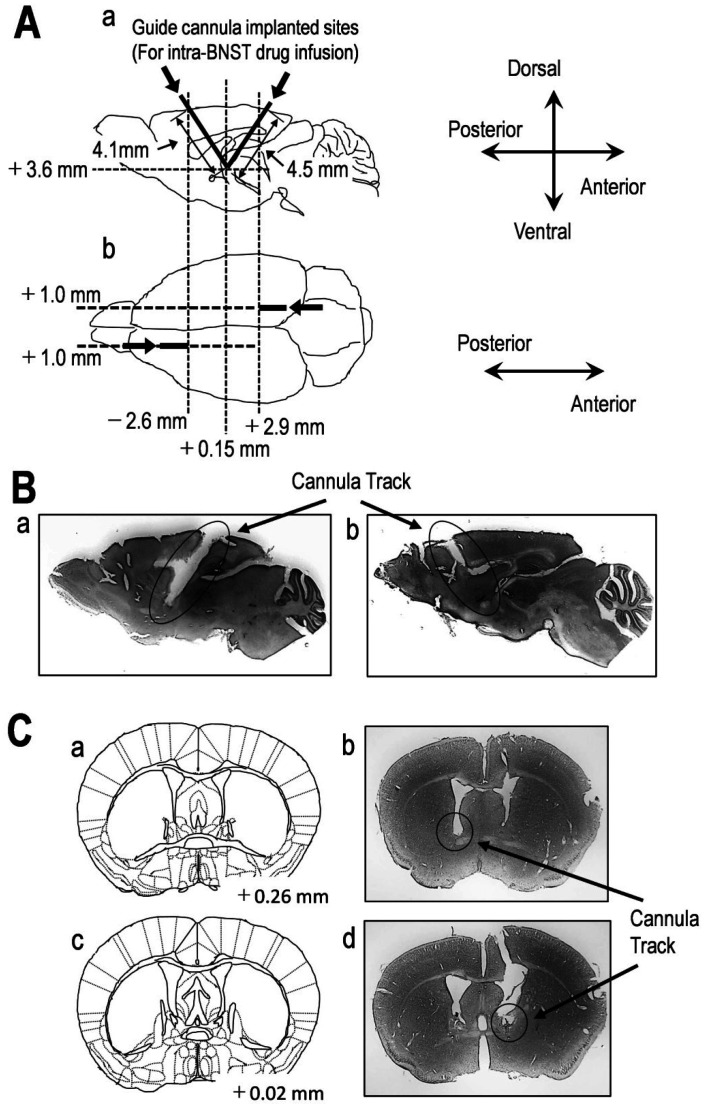
Implantation of guide cannulas into the BNST for drug microinjection.

Guide cannula implantation was performed by the methods we have previously reported [16],[27],[32],[33]. Since we implanted two guide cannular in this study, the method was modified for performing to the bilateral drug injections. For bilateral BNST drug injection in awake mice, we implanted the two guide cannulas into the BNST at least 5 days before the behavioral test. Mice were anesthetized with a mixture of medetomidine hydrochloride and butorphanol tartrate (0.3 and 5.0 mg/kg, respectively; Wako Pure Chemical Industries Ltd., Tokyo, Japan) and midazolam (4.0 mg/kg; Sandoz Ltd., Yamagata, Japan) to ensure the loss of sensation, including loss of pain sensation and immobilization during procedures. After the anesthesia, mice were placed in a stereotactic frame, and four holes were made in the skull using a dentist drill. Two holes were used for placement of steel guide cannulas (AG-8; length = 8 mm, i.d. = 0.4 mm; o.d. = 0.5 mm; Eicom, Kyoto, Japan), and the other two holes were made to anchor the stabilizing screws. For bilateral BNST injection of artificial cerebrospinal fluid (aCSF) or drug injection (Figure 1A) of freely-moving mice (without the anesthesia), two guide cannulas were implanted at the site, followed by dummy cannulas with cap nuts. However, because each cap nut had an o.d. of 5 mm, the cap nuts interfered with each other when inserted vertically. Therefore, these guide cannulas were inserted at an angle of 60° to the anterior and posterior sides, respectively (Figure 1A). These stereotaxic coordinates (mm), according to the Paxinos mouse brain atlas [34] were as follows: for intra-BNST injection from the anterior side for the right hemisphere, anteroposterior (AP): +1.6 mm, lateral (L): −0.5 mm from bregma, depth (DV): −4.1 mm; from the posterior side for the left hemisphere, AP: −3.05 mm, L: −0.5 mm from bregma, DV: −4.4 mm. Coordinates refer to the bregma and the dura surface (Figure 1A) [34],[35]. Each cannula was held in position by dental cement (GC Unifast II, GC Dental Products Corp., Tokyo, Japan) attached to the stabilizing screw. The dummy cannulas (AD-8; Eicom) were inserted into the guide cannulas and fixed with cap nuts (AC-1; Eicom) until the behavioral experiments. To reduce the pain by surgery, we applied the 5% of EMLA cream which contains the two different active local anesthetics, lidocaine and prilocaine, twice every day until behavioral experiments after the surgery. After waking from anesthesia, mice were caged until the behavioral assessment, and experiments were performed. Guide cannula-implanted mice were handled individually, once daily, for 2 days before surgery, and then twice daily during the next 4 days after anesthesia recovery until the behavioral tests were performed.

The drug was dissolved in aCSF. It has been demonstrated that the administration of a 0.1 µL volume over 1 min gave a 0.12 mm^3^ (≈ 0.493 mm × 0.493 mm × 0.493 mm) volume [36]. Microinjection was carried out by infusing 0.125 µL of drug solution (0.1 µL/1 min) using an Eicom cannula swivel unit (SSU-20) attached to an injector and a 5-µL Hamilton syringe. A dialysis probe was used for drug infusion, and the tip of the dialysis membrane part (AI-802, o.d. = 0.35 mm; Eicom) was cut and adjusted to 8 mm (the same length as the guide cannula). A polytetrafluoroethylene coiling tube (CT-20; Eicom) was used to infuse aCSF or drugs, and the mouse was allowed to move freely during drug infusion in the home cage. After drug injection, the injection probe was kept in place for at least 5 min, to minimize the spread and leaking of the drug along the injection track [37]. The drug was infused 30 min before the behavioral tests. The probe was removed 5 min after drug injection, and each mouse was kept in the home cage and monitored until the behavioral tests.

### 2.3. Verification of guide cannula placement

Histological confirmation of cannula placements in the BNST for drug microinjection was performed after the behavioral experiments. Mice were transcardially perfused with ice-cold 0.1 M phosphate-buffered saline (PBS) for 15 min, followed by filtered ice-cold 4% paraformaldehyde (PFA) in 0.1 M PBS for 15 min. The brains were carefully removed and further post-fixed in filtered 4% PFA for at least 24 h, then 30% sucrose at 4 °C for at least 48 h. Afterward, 40-µm-thick sections were cut from the frozen brain block, using a cryostat at −15 °C (Leica CM1100; Leica Biosystems, Nußoch, Germany), and mounted on poly-L-lysine-coated glass slides (Matsunami Glass Ind., Ltd., Osaka, Japan; Cat. # S7441). After washing with PBS for 5 min, the sections were incubated with Mayer's Hematoxylin Solution (Wako Pure Chemical Industries Ltd.; Cat. # 131–09665) at room temperature (25 ± 2 °C) for 5 min. The hematoxylin-stained sections were washed with 50 °C water for 5 min. Images were captured using an 8-LED USB Digital Microscope Endoscope Magnifier Camera (FB-CMXW02; Koolertron, Shenzhen, China) equipped with Micro Capture Pro software (Celestron, LLC, Torrance, CA, USA). Probe placements were verified by adapting the Paxinos mouse brain atlas [34], mainly for the dorsal and ventral BNST (Figure 1B and **C**). Mice with misplaced cannula were excluded from statistical analysis.

### 2.4. Behavioral tests

Adult male mice (8–12 weeks old) were used for the behavioral test. All behavioral tests were performed between 10:00 and 16:00 h and were conducted and analyzed by investigators blinded to the group assignments. Guide cannula-implanted mice were handled individually, once daily for 2 days before surgery, and then twice daily for 4 days after recovery from anesthesia until the behavioral tests were performed.

#### 2.4.1. Open field test

Open field tests were carried out at least 20 min before the TST and analyzed by investigators who were blinded to the group assignments. The tests were performed during the light phase (10:00–14:00 h) of the light-dark cycle. Locomotor activity was measured for 5 min by placing a mouse in the center of an acrylic plastic open field box arena (W: 294 mm × D: 294 mm × H: 297 mm) illuminated with a light bulb (40 lux). The bottom and four inner walls of the box were covered with non-reflective paper. Each mouse was habituated to the arena of the test box for 5 min (24 h before the test). Mice were placed in the center of the open field and recorded on a home video without an investigator present. Locomotor activity was scored as the number of line-crossings—when a mouse removed all four paws from one square and entered another.

Approach to placing the guide cannulas into the bed nucleus of the stria terminalis (BNST) for drug microinjection. (A) Schematic drawing indicates that the guide cannulas approached the bilateral BNST at an angle of 60° to the anterior and posterior sides (a, b). (B) Sample of cannula tracks for two guide cannulas in sagittal mouse brain sections stained with hematoxylin (a, b), showing cannula tracks above the BNST. (C) Samples of coronal sections brain sketch (a, c) and representative brain sections stained with hematoxylin (b, d), showing cannula tracks above the BNST.

#### 2.4.2. TST

All TST experiments were performed during the light phase (12:00–16:00 h) of the light-dark cycle. Each mouse was individually suspended by the tail using adhesive tape placed approximately 2 cm from the tip of the tail via two hanging hooks connected to the ceiling of the test box located 42 cm above the bench top. Each mouse was suspended for 10 min and recorded on a digital video camera in the absence of an investigator. Another investigator, who did not perform the TST experiment, measured the immobility time during the TST after the experiments by using the ANY-maze software, so that the investigators remained blinded to the allocated groupings. Immobility duration was measured as the sum of the time-periods during which the mouse was motionless for, at least, 2 s. Immobile behavior sensitivity was set at 70%, and the mouse needed to be immobile for 1 s to initiate scoring of immobility. After the TST, mice were returned to their respective home cages [38]. As an index of learned despair, 10 min of the test session was divided into two sections; the initial 5 min (0–5 min) and the final 5 min (5–10 min), respectively, and 30 min after the bilateral intra-BNST injection of aCSF or drugs, the mice were subjected to two sets of TST with an intertrial interval of 24 h.

### 2.5. Chemicals and reagents

LPS (Cat. # L3129, serotype O127:B8) and MDL-12,330A hydrochloride (Cat. # M182) were purchased from Sigma-Aldrich (St. Louis, MO, USA). Neomycin sulfate was purchased from Tokyo Chemical Co., Ltd. (Cat. # F0649, Tokyo, Japan), and 1-naphthylacetyl spermine (Naspm) hydrochloride was purchased from Cayman Chemical Co. (Cat. # 18453, Ann Arbor, MI, USA).

### 2.6. Statistical analysis

**Figure 2.**
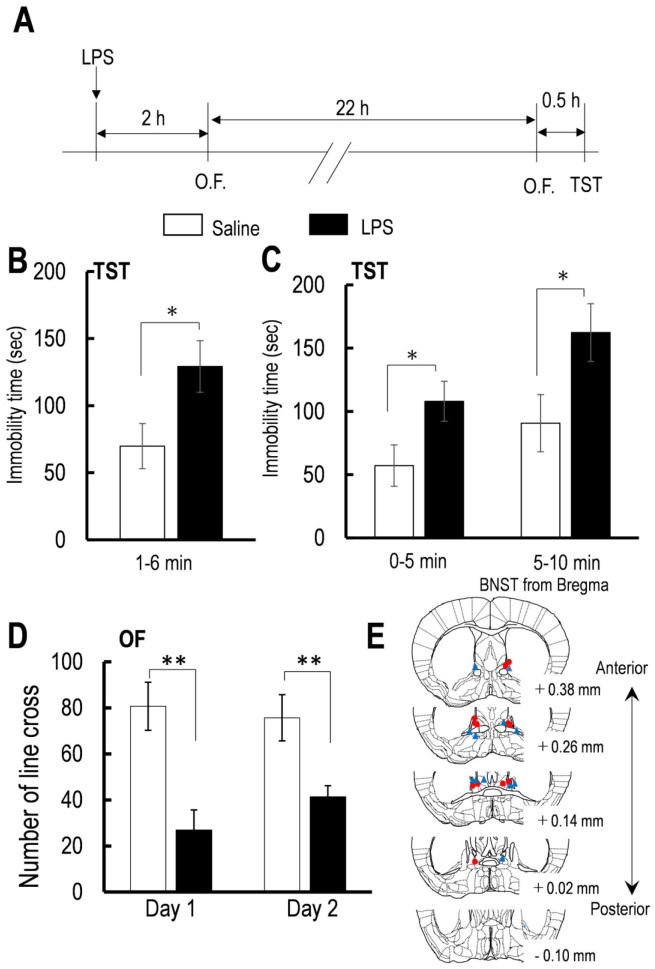
The effects of LPS on the immobility time during TST and locomotor activity.

**Figure 3.**
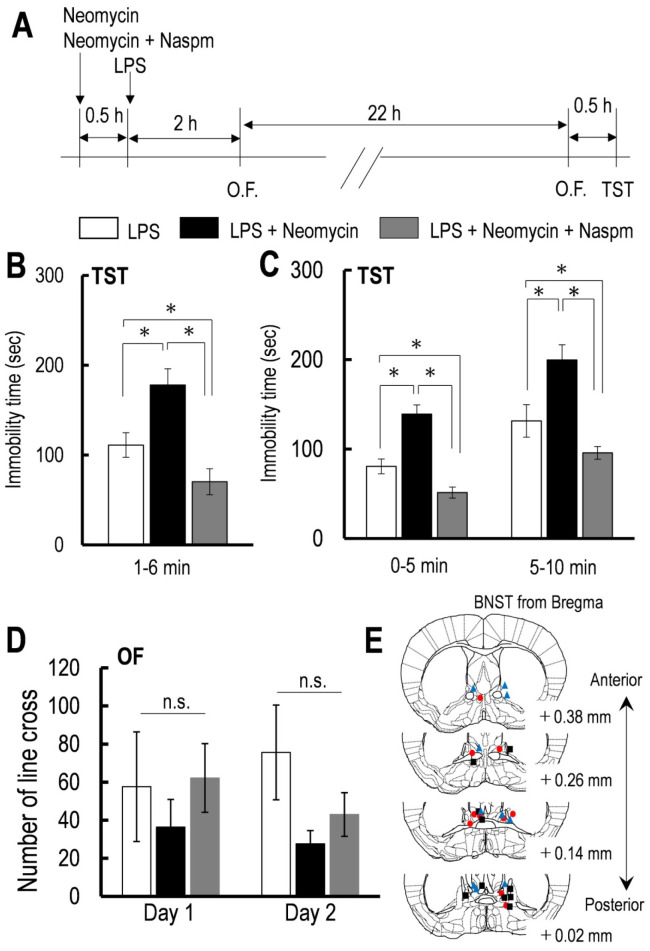
The effects of sole application of neomycin and co-application of neomycin with Naspm on the LPS-increased immobility time during TST and locomotor activity.

**Figure 4.**
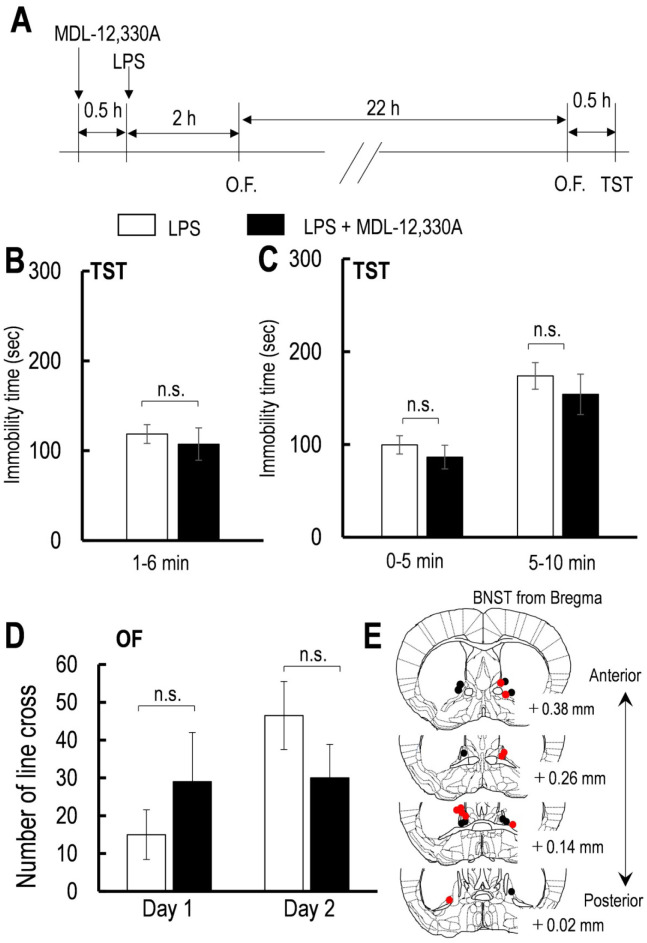
The effects of MDL-12,330A on the LPS-increased immobility time during TST and locomotor activity.

Student *t*-tests were used for comparisons between groups in Figure 2B, 3B, and 4B. One-way analysis of variance (ANOVA) was performed, followed by Bonferroni post hoc test, as appropriate for Figure 3B. Two-way repeated-measures ANOVA was performed to assess the effect of treatment in Figure 2C, 2D, 3C, 3D, 4C, and 4D, followed by one-way ANOVA with paired *t*-test for Figure 2C, 2D, 4C, and 4D or Bonferroni post hoc test between groups for Figure 3C and D. All statistical analyses were performed using EZR (Easy R) software (version 1.38; Saitama, Japan) [39]. All data in the bars indicate the mean ± standard error of the mean (SEM). All analyses were set at *p* < 0.01 (**) or *p* < 0.05 (*).

## 3. Results

### 3.1. LPS-induced despair-like behavior

LPS causes the depressive-like behaviors, which include behavioral despair and anhedonia in rodents. The TST and forced swimming test (FST) are both despair-based assays and their immobility time during the TST or FST is used as a measure of behavioral despair level. When the mice realize there is no escape, the duration of immobility is increased, particularly in the second rather than first half of the test session during the TST and FST, indicating that TST and FST are both conceptually similar to the “learned helplessness” exhibited by naïve rodents [40]. It is thus called “learned despair”. In general, rodents treated with LPS display an increase in the immobility time even in the first half of the test session because of the low motivation to overcome the aversive situation [14],[16],[32].

Although the concept of TST and FST is similar, performing the tests using monoamine transporter knockout mice has suggested that the TST is dependent on both noradrenaline and serotonin, contrary to the FST, which is mainly dependent on serotonin [32],[41]. Considering this subtle difference between the TST and FST, we performed the TST for 10 min to investigate whether the immobility time is mainly due to low motivation or learned despair in the first and last 5 min, not only the results for 5 min from 1 to 6 min. There was an increase in the immobility time for the 5 min from 1 to 6 min in 24 h (*p* < 0.05, Figure 2B). Although, Two-way repeated measures ANOVA revealed no significant LPS × time interaction effect on the immobility time throughout the test period [*F* (1,10) = 1.65, *p* = 0.228, Figure 2C], the individual factor (LPS or Time) was significant [LPS: *F* (1,10) = 5.28, *p* = 0.0448; Time: *F* (1,10) = 29.32, *p* = 0.00029; Figure 2C]. The paired *t*-test indicated that the immobility time was longer in mice injected with LPS compared with saline (which served as a control) in both the first and last 5 min (0–5 min: *p* < 0.05; 5–10 min: *p* < 0.05; Figure 2C). These results indicated that mice treated with LPS exhibited not only the learned behavioral despair but also low motivation to overcome the aversive situation. Two-way repeated-measures ANOVA revealed no significant LPS × time interaction on the locomotor activity [*F* (1,10) = 2.24, *p* = 0.166, Figure 2C]. However, as individual factors, LPS, but not the Time, markedly affected the locomotor activity [LPS: *F* (1,10) = 17.1, *p* = 0.0020; Time: *F* (1,10) = 0.53, *p* = 0.482]. The paired *t-*test indicated that LPS decreased the locomotor activity 2 and 24 h after the LPS injection (2 h, *p* < 0.01; 24 h, *p* < 0.01; Figure 2D).

Figure 2 (A) Schedule of LPS injection and behavioral test. (B) Effect of LPS on the immobility time during TST for 5 min from 1 to 6 min (saline, n = 6, LPS, n = 6). (C) Effect of LPS on the immobility time during TST for first and last 5 min (saline, n = 6, LPS, n = 6). (D) Effect of LPS on the locomotor activity at 4 and 24 h after LPS injection (saline, n = 6, LPS, n = 6). (E) Schematic of mice coronal section showing placement of the guide cannula for intra-BNST injection of aCSF for each saline (red circle) and LPS mouse group (blue triangle). All data are the mean ± SEM. n = number of mice. The effects of drug administration reaching statistical significance (**p < 0.01, *p < 0.05) are noted. ns. represents not significant.

### 3.2. The effect of Gαq protein signaling on the LPS-induced despair-like behavior during TST

To investigate whether the Gαq protein signaling in the BNST effect on the LPS-induced despair-like behavior, the rats received an intra-BNST injection of neomycin (6.5 mM) [42], a blocker of phospholipase C, which can be activated by the Gαq protein-coupled receptors [43]. In addition, we performed intra-BNST injection of Naspm (3 mM) [44], a specific inhibitor of Ca^2+^-permeable AMPA receptors, to mimic the final form of α_1_-adrenergic receptor stimulation because the α_1_-adrenergic receptor stimulation activates ADAR2, which increases the Ca^2+^-impermeable AMPA receptors [21], resulting in the RNA editing of AMPA receptor subunit GluR2 [26]. Although the Naspm-sensitive GluR2-lacking Ca^2+^-permeable AMPA receptors are predominantly expressed in the parvalbumin-positive GABAergic neurons, the BNST lacks parvalbumin-positive GABAergic neurons [45]. Therefore, this property enables determining the outcome of intra-BNST injection of Naspm.

One-way ANOVA revealed that the neomycin and Naspm significantly affected the immobility time during the TST throughout the test period [*F* (2,12) = 8.63, *p* = 0.0048, Figure 3C]. Bonferroni post hoc test indicated a significantly longer immobility time in mice that received intra-BNST injection of neomycin compared with aCSF (neomycin vs. aCSF: *p* =0.022, Figure 3B). Conversely, co-pretreatment with intra-BNST injection of neomycin and Naspm restored the neomycin-induced increase in the immobility time. Its immobility time was shorter than that of mice that received intra-BNST injection of aCSF (neomycin + Naspm vs. neomycin: *p* < 0.0042; neomycin + Naspm vs. aCSF: *p* < 0.035, Figure 3B). Two-way repeated-measures ANOVA revealed a significant drug (neomycin and Naspm) × time interaction effect on the immobility time [*F* (2,12) = 5.71, *p* = 0.018, Figure 3C]. One-way ANOVA revealed that at each time point of both 0–5 and 5–10 min, drug treatment affected the immobility time during the TST [0–5 min: *F* (2,12) = 6.68, *p* = 0.011; 5–10 min: *F* (2,12) = 11.88, *p* = 0.0014; Figure 3C]. Bonferroni post hoc test indicated a significantly longer immobility time in mice pretreated with intra-BNST injection relative to the control (aCSF) in both the first and last 5 min of the 10-min TST (neomycin 0–5 min: *p* < 0.05 vs. aCSF 5–10 min: *p* < 0.05 vs. aCSF; Figure 3C). In addition, the immobility time in mice pretreated with intra-BNST injection of neomycin and Naspm was significantly lower than aCSF-pretreated mice in both the first and last 5 min of the 10-min TST (neomycin + Naspm 0–5 min: *p* < 0.05 vs. neomycin 5–10 min: *p* < 0.01 vs. aCSF; neomycin + Naspm 0–5 min: *p* < 0.05 vs. aCSF 5–10 min: *p* < 0.05 vs. aCSF; Figure 3C). Two-way repeated-measures ANOVA revealed no significant treatment × time interaction effect affected on the locomotor activity [*F* (2,12) = 1.49, *p* = 0.166, Figure 3C], indicating that these drugs did not affect the LPS-induced decrease in locomotor activity. These results indicated that Gαq protein signaling not only prevents the LPS-induced despair-like behavior, which is due to the low motivation to overcome the aversive situation, but also the learned behavioral despair.

Figure 3 (A) Schedule of LPS injection and behavioral test. (B) Effect of neomycin and neomycin with Naspm on the LPS-increased immobility time during TST for 5 min from 1 to 6 min (aCSF: n = 5, neomycin: n = 6, neomycin + Naspm: n = 5). (C) Effect of neomycin and neomycin with Naspm on the LPS-increased immobility time during TST for the first and last 5 min (aCSF: n = 5, neomycin: n = 6, neomycin + Naspm: n = 5). (D) Effect of neomycin and neomycin with Naspm on the LPS-induced lower locomotor activity at 4 and 24 h after LPS injection (aCSF: n = 5, neomycin: n = 6, neomycin + Naspm: n = 5). (E) Schematic of mice coronal section showing placement of the guide cannula for intra-BNST injection of aCSF (red circle), neomycin (blue triangle) and neomycin with Naspm (black square) for LPS-treated mice. All data are the mean ± SEM. n = number of mice. The effects of drug administration reaching statistical significance (*p < 0.05) are noted. ns. represents not significant.

### 3.3. The effect of Gαs protein signaling on the LPS-induced despair-like behavior during TST

We further investigated whether the Gαs protein signaling affects the LPS-induced despair-like behavior. We performed intra-BNST injection of MDL-12,330A (10 µM) [3], a blocker of adenylyl cyclase, which is activated by Gαs protein-coupled receptor stimulation, 30 min before LPS injection.

The intra-BNST injection of MDL-12,330A did not affect the LPS-induced increase in the immobility time during the first 5 min (from 1 to 6 min) of the TST (*p* = 0.301, Figure 4B). Two-way repeated-measures ANOVA revealed no effect of the intra-BNST injection of MDL-12, 330A on the LPS-induced increase in the immobility time during the TST [*F* (1,10) = 0.096, *p* = 0.76, Figure 4C]. Two-way repeated-measures ANOVA showed no significant treatment × time interaction effect on the locomotor activity [*F* (1,12) = 2.201, *p* = 0.18, Figure 2C], thereby suggesting that these drugs did not affect the LPS-induced decrease in locomotor activity. These results indicated that the Gαs protein signaling in the BNST does not regulate the LPS-induced despair-like behavior.

Figure 4 (A) Schedule of LPS injection and behavioral test. (B) Effect of MDL-12, 330A on the LPS-increased immobility time during TST for 5 min from 1 to 6 min (aCSF, n = 7, MDL-12, 330A, n = 5). (C) Effect of MDL-12, 330A on the LPS-increased immobility time during TST for the first and last 5 min (aCSF, n = 7, MDL-12, 330A, n = 5). (D) Effect of MDL-12, 330A on the LPS-induced lower locomotor activity at 4 and 24 h after LPS injection (aCSF, n = 7, MDL-12, 330A, n = 5). (E) Schematic of mice coronal section showing placement of the guide cannula for intra-BNST injection of aCSF (red circle) and MDL-12, 330A (black circle) for LPS-treated mice. All data are the mean ± SEM. n = number of mice.

## 4. Discussion

The present study demonstrated that the blockade of Gαq protein-activated phospholipase C in BNST by neomycin abrogated the LPS-induced despair-like behavior. Furthermore, the mimic of the final form of α_1_-adrenergic receptor stimulation in BNST by Naspm to block the Ca^2+^-permeable AMPA receptor in BNST restored the aggravated effect of neomycin and partly prevented the LPS-induced despair-like behavior. These results suggested that the Gαq protein-coupled receptor stimulation in BNST can prevent the induction of inflammation-associated depression. However, we previously reported that the LPS-induced depressive-like behaviors are associated with α_1_-adrenergic receptor stimulation-induced down-regulation of the plasma membrane protein level of AMPA GluR1 in the ventral tegmental area (VTA) and medial prefrontal cortex (mPFC) [16]. Both regions are known as the reward system in the brain. Here, we discussed the role of Gαq protein-coupled receptors within the BNST on the induction of depressive-like behavior and the contradiction obtained in our research studies.

Our previous study demonstrating the contribution of α_1_-adrenergic receptor stimulation-induced down-regulation of the plasma membrane protein level of AMPA GluR1 on LPS-induced depressive-like behavior in rats occurred in response to intracerebroventricular injection of prazosin in rats, and we investigated only the VTA and mPFC [16]. The BNST neurons receive noradrenergic innervation from the A1 and A2 cell groups in the nucleus of the solitary tract [46]. Contrariwise, locus coeruleus noradrenergic neurons innervate the dopaminergic neurons in the VTA [47]. VTA neurons innervated by the BNST GABAergic neurons, which are depolarized by α_1_-adrenergic receptor stimulation [48], indicates that the α_1_-adrenergic receptors within the BNST exert inhibitory regulation of the VTA neuronal activity. In contrast, the α_1_-adrenergic receptors in the VTA produce the disinhibition within the VTA by inhibiting the presynaptic GABA release [49]. The α_1_-adrenergic receptors in the VTA are highly expressed postsynaptically on dopaminergic neurons [50],[51]. Taken together, the activation of α_1_-adrenergic receptors within the VTA produces the excitatory effect, but the stimulation of α_1_-adrenergic receptors within the BNST negatively regulates the dopaminergic neurons in the VTA.

Activation of α_1_-adrenergic receptors induces LTD in the visual cortex, hippocampus, VTA, and BNST [16],[21],[52],[53],[54],[55], which is dependent on the NMDA receptor activation in the visual cortex [49],[55] and hippocampus [53]. Down-regulation of membrane-bound AMPA-GluR1 in the VTA is shown to be induced by α_1_-adrenergic receptors [16]. In addition, an RNA editing-induced increase in the Ca^2+^-impermeable AMPA receptor-elicited LTD has been reported in the BNST [21]. Mechanistically distinct forms of Gαq protein-coupled receptor-induced LTD in the BNST have been suggested between mGluR5 and α_1_-adrenergic receptors [21]. In the present study, Naspm partly prevented the LPS-induced despair-like behavior, indicating that stimulation of the α_1_-adrenergic receptors may prevent the induction of inflammation-associated depression. In the hippocampus, LPS suppresses the induction of both long-term potentiation and LTD by blocking the NMDA receptor-mediated Ca^2+^ influx [56]. LPS induces numerous Fos-positive neurons in the BNST [57] and activates the A1 and A2 neurons [58]. However, it remains unclear whether activation of the α_1_-adrenergic receptors is involved in the LPS-induced neuronal activation in the BNST. Our results in this study indicated that activation of α_1_-adrenergic receptor-related Gαq protein signaling and the LTD induced by increasing the Ca^2+^-impermeable AMPA receptors, which is due to the RNA editing of GluR2, may contribute to preventing LPS-induced despair-like behavior. It implies that the LPS-induced despair-like behavior may require the inhibition of α_1_-adrenergic receptor-related Gαq protein signaling and an increase in the Ca^2+^-impermeable AMPA receptors.

Stress is associated with increases in released noradrenaline [59], and α_2_-adrenergic receptor activation is anxiolytic because of the inhibition of noradrenaline release via Gi protein signaling [60]. Serotonin and noradrenaline reuptake inhibitors (SNRI) improve treatment outcomes in patients with MDD compared with SSRI [61], indicating that noradrenaline is important for MDD treatment. SNRI has the therapeutic lag-time from the initiation of antidepressant and the onset of its action [62]. Chronic treatment with SNRI, but not SSRI, down-regulates the β-adrenergic receptors in some regions of the brain [63]. By contrast, the α_1_-adrenergic receptor is up-regulated by chronic treatment with an antidepressant [64]. The α_1_-adrenergic receptors-mediated Gαq protein signaling within the BNST is involved in the anxiety-like behavior through RNA editing of the GluR2 subunit of the Ca^2+^-impermeable AMPA receptor, which results in LTD [21]. On the other hand, another Gαq protein-coupled receptor is 5-HT_2C_ serotonin receptors deleted mice exhibit the anxiolytic phenotype through a selective blunting of BNST corticotropin-releasing hormone neuronal activation in response to anxiety stimuli [65]. In the present study, the mimicking by Naspm to block specifically the Ca^2+^-permeable AMPA receptor improve the neomycin-aggravated LPS-induced despair behavior, indicating that the α_1_-adrenergic receptors-mediated Gαq protein signaling-produced RNA editing of the GluR2 subunit of the Ca^2+^-impermeable AMPA receptor within the BNST. It might be beneficial for MDD that the combination of SNRI with the drug which block the Ca^2+^-permeable AMPA receptor within the BNST until the SNRI exert the antidepressant effect, along with the up-regulation of α_1_-adrenergic receptor and the down-regulation of β-adrenergic receptors in brain. However, contradictory data have been reported and involve brain regions other than the BNST. Despite that the mechanisms of induction of MDD and the therapeutic mechanisms are not shared completely, the role of the Gαq protein-coupled receptor in the BNST in the etiology of MDD should be a focus in future research.

Tips of the implanted two guide cannulas were located throughout the BNST in the pharmacological experiment of this study. Therefore, the applied drugs in this study affected to whole area of BNST. However, there two distinct subdivision of highly expressed Gαq protein-coupled receptor, ex, α_1_-adrenergic receptors, the oval nucleus of dorsal BNST and the fusiform nucleus of ventral BNST located in anterior region [66]. The neurons in oval nucleus of BNST, particularly GABAergic neurons project to the central nucleus of amygdala, ventral tegmental area and the lateral hypothalamus which integrates the information about mood, particularly anxiety-like behavior [67],[68]. In contrast, the neurons in fusiform nucleus of BNST project to the central nucleus of amygdala, paraventricular nucleus, nucleus accumbens, peri-aqueductal gray, which is involved in pain [69] and reticular nuclei, which is involved in attention [68]. Therefore, the applied drugs in the present study affected not only the despair-like behavior, but also mood, anxiety, pain and attention etc. It should be investigated in the future that the role of Gαq protein signaling-produced RNA editing of the GluR2 subunit of the Ca^2+^-impermeable AMPA receptor in each subdivision of BNST.

## 5. Conclusion

We investigated the role of Gαq protein signaling within the BNST in the LPS-induced despair-like behavior. Blockade of phospholipase C-related Gαq protein signaling in the BNST aggravated the LPS-induced despair-like behavior, and blockade of the Ca^2+^-permeable AMPA receptor in BNST by Naspm abolished the aggravated effect of phospholipase C blocker. Furthermore, blockade of the Ca^2+^-permeable AMPA receptor in BNST abrogated the LPS-induced despair-like behavior. Naspm mimics the final form of the Gαq protein-coupled α_1_-adrenergic receptor, which leads us to suggest that the Gαq protein-coupled receptor stimulation-induced RNA editing of GluR2 subunit of AMPA receptor-dependent LTD within the BNST regulates the LPS-induced despair-like behavior.
